# Explaining L2 Lexical Learning in Multiple Scenarios: Cross-Situational Word Learning in L1 Mandarin L2 English Speakers

**DOI:** 10.3390/brainsci12121618

**Published:** 2022-11-25

**Authors:** Paola Escudero, Eline A. Smit, Karen E. Mulak

**Affiliations:** 1 The MARCS Institute for Brain, Behaviour, and Development, Western Sydney University, Penrith, NSW 2751, Australia; 2 Australian Research Council Centre of Excellence for the Dynamics of Language, Canberra, ACT 2601, Australia; 3 Department of Linguistics, University of Konstanz, 78457 Konstanz, Germany

**Keywords:** cross-situational word learning, L1 mandarin L2 english, minimal and non-minimal word pairs, acoustic cues, language modes, L2LP model

## Abstract

Adults commonly struggle with perceiving and recognizing the sounds and words of a second language (L2), especially when the L2 sounds do not have a counterpart in the learner’s first language (L1). We examined how L1 Mandarin L2 English speakers learned pseudo English words within a cross-situational word learning (CSWL) task previously presented to monolingual English and bilingual Mandarin-English speakers. CSWL is ambiguous because participants are not provided with direct mappings of words and object referents. Rather, learners discern word-object correspondences through tracking multiple co-occurrences across learning trials. The monolinguals and bilinguals tested in previous studies showed lower performance for pseudo words that formed vowel minimal pairs (e.g., /dit/-/dɪt/) than pseudo word which formed consonant minimal pairs (e.g., /bɔn/-/pɔn/) or non-minimal pairs which differed in all segments (e.g., /bɔn/-/dit/). In contrast, L1 Mandarin L2 English listeners struggled to learn all word pairs. We explain this seemingly contradicting finding by considering the multiplicity of acoustic cues in the stimuli presented to all participant groups. Stimuli were produced in infant-directed-speech (IDS) in order to compare performance by children and adults and because previous research had shown that IDS enhances L1 and L2 acquisition. We propose that the suprasegmental pitch variation in the vowels typical of IDS stimuli might be perceived as lexical tone distinctions for tonal language speakers who cannot fully inhibit their L1 activation, resulting in high lexical competition and diminished learning during an ambiguous word learning task. Our results are in line with the Second Language Linguistic Perception (L2LP) model which proposes that fine-grained acoustic information from multiple sources and the ability to switch between language modes affects non-native phonetic and lexical development.

## 1. Introduction

Learning a second language (L2) in adulthood is difficult. Adults typically require additional time and exposure to their target L2 compared to younger learners to reach native-like proficiency (e.g., [1,2]), and commonly exhibit prolonged difficulty with pronunciation [3,4,5,6], and lexical access [7,8]. Most researchers agree that these difficulties stem in part from differences between learners’ L1 and L2 systems. In the realm of speech learning, whether an L2 phoneme contrast is easy or difficult for a learner to perceive highly depends on how L1 speech sounds compare to those in the target L2, as advocated by most cross-language and L2 speech learning models (e.g., [9,10,11,12]). For instance, the Second Language Linguistic Perception model (L2LP; [11,12,13,14]) describes possible L2 learning scenarios depending on the acoustic proximity and overlap between L1 and L2 phoneme categories. Within the L2LP model, when two phonemes of an L2 contrast are acoustically closest to a single L1 category, learners face a NEW scenario, and have difficulty perceiving the difference between the “new” L2 contrast (e.g., English /i/-/ɪ/ for L1 Catalan, Japanese, Mandarin, Polish, Portuguese, or Russian speakers, cf. (for a review see [13]). Learners face a SIMILAR scenario when two L2 categories are close matches of an acoustically similar L1 contrast (e.g., English /æ/-/ε/ for L1 Spanish speakers or English /i/-/ɪ/ for L1 Japanese speakers, cf. [14]). In this case, learners can replicate their existing L1 categories in the L2 but adjust their boundary as needed so that they match the L2 contrast. The SIMILAR scenario is suggested to be less problematic for the L2 learner since, unlike the NEW scenario, it does not require creating new phonological categories [12,14,15].

Non-native vowel perception studies support the claim that differences in the acoustic realization of L1 and L2 phonemes influence L2 speech perception (e.g., [16,17]). For instance, Alispahic and colleagues [17] tested Australian English (AusE) and Peruvian Spanish native speakers’ discrimination of Dutch vowel contrasts. They made predictions about which Dutch contrasts would be easy and difficult for AusE monolinguals to discriminate and which would be easy and difficult for Peruvian Spanish monolinguals based on the unique acoustic relationships between L1 and L2 categories for each language. Indeed, they found that performance matched these predictions for both groups of speakers, supporting the idea that acoustic properties impact listeners’ perception of non-native sounds (see [18]) and that listeners use perceptual cues from their L1 when categorizing non-native contrasts.

L2LP additionally proposes that the difficulties and relative ease in perceiving certain L2 contrasts based on the L1–L2 acoustic relationship extend to word learning and recognition [11,12,13,19,20,21,22,23]. Specifically, L2 learning of word pairs differing in a single phonological category—also known as minimal pairs—is predicted and explained by L2 learners’ perceptual difficulty, which in turn is based on the acoustic comparisons of L1 and L2 categories (e.g., [15,20,21]. For instance, [20] tested learning of Dutch minimal and non-minimal pairs in L1 Spanish speakers learning Dutch. Word pairs were separated into easy and difficult categories based on whether these contrasts exist in Spanish and are thus NEW for learners and predicted to be difficult, such as /i-ɪ/—Spanish only has /i/—or whether the L2 contrasts are similar to Spanish and predicted to be easy, such as /ɪ-a/. In contrast with Dutch native speakers who performed equally well for all minimal word pairs, the L1 Spanish speakers performed worse for the difficult minimal pairs compared to the easy minimal pairs, confirming the L2LP proposal that L2 perceptual difficulty influences L2 lexical representations and L2 word learning.

In the present study, we examined L1 Mandarin L2 English learners’ ability to learn English words in an ambiguous cross-situational word learning paradigm (CSWL) containing English sounds that do not exist in their L1 and compared their performance to English monolinguals. Specifically, we tested whether these L2 learners (a) perform equally or worse than English monolinguals in ambiguous word learning situations and if so, whether (b) their performance on minimal pairs may be explained by the relationship between L1 and L2 vowels and consonants, as proposed by most L2 speech learning models, including the L2LP. CSWL paradigms resemble a common real-world word learning situation in which word-objects pairings are presented ambiguously, in the context of other candidate pairings. Across multiple encounters with the words and items, learners can derive the correct word-object pairing through bottom-up statistical tracking mechanisms (e.g., [24]) or top-down hypothesis testing mechanisms (e.g., [25]).

Previous studies have demonstrated that both simultaneous bilinguals and L2 learners (also known as sequential bilinguals) can learn the pseudo words we present in a statistical word learning task. We define simultaneous bilinguals as learners who were exposed to two languages from birth and sequential bilinguals as L2 learners with exposure to the L2 after acquiring a first language, with onset of L2 acquisition during childhood, adolescence or adulthood. In the case of our study, all learners were exposed to English as a foreign language at school and therefore are referred to as L2 learners or sequential bilinguals. While most simultaneous bilinguals acquire proficiency comparable to monolingual speakers of their two languages, L2 learning can yield different levels of proficiency, in the case of the present group and those tested in [26]. The participants in both groups tested here followed university education in English, thus their L2 proficiency was advanced enough to understand English at a tertiary education level. When learning English words in a CSWL task Singaporean English-Mandarin simultaneous bilinguals had higher word-learning accuracy than monolingual English speakers [27]. In contrast, no difference in CSWL between highly proficient L2 English speakers with heterogeneous L1 backgrounds and monolingual English listeners was found in [26]. In both CSWL studies, participants were tested on their ability to learn eight words, four of which differed from one another on their initial consonant, forming consonant minimal pairs (cMP; e.g., BON-TON), and four of which formed vowel minimal pairs (vMP; e.g., DIT-DUT). Pairing a word from one set with one from the other set formed a non-minimal pair (nonMP; e.g., BON-DIT). Even though the simultaneous bilinguals in [27] outperformed monolinguals in accuracy, their reaction time for vMPs was slower to that of monolinguals. This may have been due to difficulties distinguishing the words DIT (/dɪt/) and DEET /dit/, as the vowel /ɪ/ is not found in the Mandarin vowel inventory. Alternatively, a vowel bias in Mandarin could have impacted reaction time, as vowels appear to provide stronger lexical identity in Mandarin compared to consonants [28], unlike in English. This may result in delayed processing of words differing in a single vowel by English-Mandarin bilinguals. The contrasting findings between learner groups, namely bilinguals in [27] versus L2 learners in [26], may be due to their specific linguistic background or to the English variety of the stimuli (American versus Australian English).

We presented L1 Mandarin L2 English learners with the same CSWL task as in [26,27] including the same eight words (i.e., /dit/, /dɪt/, /dʊt/, /dut/, /bɔn/, /pɔn/, /tɔn/ and /dɔn/, and pairings (i.e., nonMPs, vMPs and cMPs), compared their performance to that of AusE monolinguals, and assessed whether the relationship between Mandarin and English vowels and consonants can explain L2 word learning. First, we expected our AusE monolinguals to perform similarly to those tested in [27], with higher performance for nonMPs and cMPs compared to vMPs. In contrast, we expected our L1 Mandarin L2 English learners to have more L1 interference and have less optimal L2 representations than simultaneous bilinguals and therefore predicted they would find vMPs more difficult than cMPs or nonMPs, with their high L2 proficiency leading to similar performance to AusE monolinguals in cMPs and nonMPs. This prediction is in line with the L2LP model and with many other cross-language and L2 speech learning models. Specifically, if L1 Mandarin L2 English learners continue to have L2 representations that are L1-like (as shown in [12]), English words containing the vowels /ɪ/, /ʌ/, /ʊ/ should be particularly difficult to master [29], as these vowels are not found in Mandarin and are acoustically very similar to Mandarin /i/, /a/, /u/, leading to a NEW scenario that has not been resolved despite advanced L2 proficiency. Although many previous studies have shown plasticity for L2 learners in the phonetic/phonology domain, L2 proficiency does not seem to have a clear correlation with mastering new contrasts [13]. Conversely, previous studies have shown English consonants appear to be easier to perceive for Mandarin speakers, suggesting that they constitute a SIMILAR scenario, leading to better L2 performance from the start [30,31].

Finally, we tested a developmental tenet of L2LP which poses that transition from naïve listening to high L2 proficiency results in L2 perceptual and lexical development, such as creating or shifting of phonological categories to better represent the L2 [11,12]. Previous results have been mixed, as no difference between naïve Spanish-speaking listeners and those who had been learning Dutch in an immersive environment has been found [13,20], while some L2 perception studies report a positive effect of L2 experience [32,33] and others find no effect [13,34,35]. L2 immersion in a city where the L2 is spoken is a further opportunity to learn and be surrounded by the L2 in daily life, as opposed to only in a classroom. Within the L2LP framework, language immersion is seen as richer and more impactful language exposure, which should lead to further learning and in turn to higher L2 proficiency. Thus, if immersive experience with the specific target L2, namely AusE, influences performance, L2 word learning accuracy will be higher for L1 Mandarin L2 English learners who are immersed in the target L2 in Sydney, Australia than those who live in their home country (Shanghai, China). Our study therefore differs from previous studies in examining a homogeneous group of L2 learners who have Mandarin as their L1.

## 2. Materials and Methods

### 2.1. Participants

Sixty participants took part in the experiment. Thirty-one were AusE monolinguals from Sydney (*M*_age_ = 26, 21 females, 10 males), and 29 were L1 Mandarin L2 English participants who were divided in two groups according to their place of residence during testing: 11 lived in Sydney, Australia (MandSyd, *M*_age_ = 27.34, 9 females, 2 males) and 18 in Shanghai, China (MandShanghai, *M*_age_ =22, 10 females, 8 males). Participants tested in Sydney were undergraduate psychology students or people from the local community recruited through word-of-mouth, advertisements or the university’s participant recruitment system. Mandarin speakers in Sydney were native in Mandarin and indicated to speak and understand (Australian) English at advanced to native level. Participants tested in Shanghai were recruited through word-of-mouth and were all native Mandarin speakers at East China Normal University. They had studied English for an average of 14 years. They used Mandarin-Chinese daily, and English occasionally at university. Specific data regarding the precise number of years of English experience per participant was not collected or is no longer available. Participants received course credit or $10 travel compensation for their participation. Written informed consent was obtained from all participants prior to the start of the experiment, and the study was approved by the Western Sydney University Human Research Ethics Committee.

### 2.2. Stimuli

#### 2.2.1. Pseudo Spoken Words

Stimuli consisted of eight monosyllabic pseudo words recorded by a female speaker of AusE using infant-directed speech (IDS). The words originate from a prior CSWL study [36] and have been used in other word learning and CSWL studies [26,27,37,38,39,40] with no effect of item on word learning accuracy. For the present study, we chose to use the same IDS stimuli in order to directly compare our results to previous studies, which were aimed at testing word learning in infants versus adults. We also chose IDS stimuli because many previous studies have shown that IDS facilitates word learning in infants learning their native language [41,42] and adult second language learners [43,44,45,46].

Words followed a CVC structure following English phonotactics. Per word, two tokens were selected to match prosodic contours across all words, with one token having a rising prosodic contour whereas the second has a descending prosodic contour. Four words differing from one another on their initial consonant formed consonant minimal pairs and followed a /Cɔn/ structure (cMP; e.g., BON-TON). The other four words followed a /dVt/ structure, forming vowel minimal pairs (vMP) with one another (e.g., DIT-DUT). Pairing a word from one set with one from the other set formed a non-minimal pair (nonMP; e.g., BON-DIT).

#### 2.2.2. Pseudo Visual Referents

**Figure 1:**
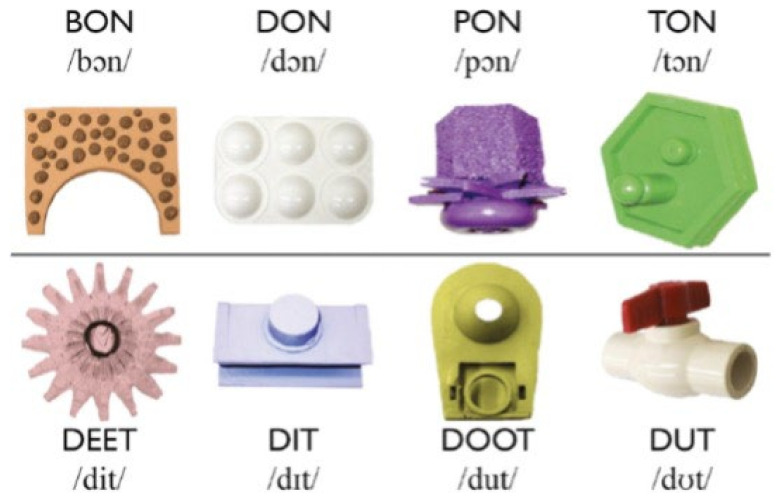
The eight pseudo words and their visual referents. The four words in the top row are minimally different in their initial consonant, whereas the words on the bottom are minimally different in their vowel. The vowel used for the consonant minimal pairs is /ɔ/ as in POT. Vowels used for the vowel minimal pairs are /i/ as in BEAT, /ɪ/ as in BIT, /u/ as in BOOT, and /ʊ/ as in PUT. Figure 1 originates from [27].

Each word was paired with a visual referent, which consisted of colour pictures of pseudo items (see Figure 1). These pictures have been used in prior CSWL studies (e.g., [23,26,27,36,40,47]. Pictures were 210 × 206 pixels.

### 2.3. Procedure

After obtaining written consent, participants filled out a language background questionnaire. They then completed the CSWL task, comprising a learning phase followed by a test phase. For this, participants were seated in front of a laptop computer with a 17-inch monitor and were asked to wear headphones throughout the experiment. The experiment was run using the software package E-prime (version 2.0, Psychology Software Tools Inc., Sharpsburg, PA, USA).

The learning phase consisted of 36 trials (as in [26]), with each word-referent pairing presented nine times. As in the previous CSWL studies, participants were instructed to look at the images and listen to the sounds but were not informed that this was a word learning experiment. During each trial, two visual referents were presented on the screen on a white background, centered vertically. After the images had been on the screen for 500 ms (to keep the experimental design consistent with prior CSWL studies [26,27,38,40]), the auditory labels corresponding to each referent were played such that the referents were named left-to-right or right-to-left with 500 ms between tokens, without indication of which label belonged to which referent. Trials were randomized for each participant and were controlled to ensure that each image was presented simultaneously with every other image at least once and at most twice (for more specific details regarding the counterbalancing of the trials, see [26]). In total, there were 24 nonMPs pairs, 6 cMP pairs, and 6 vMPs. Trials lasted for 3.5 s leading to a total learning phase of approximately 3 min. Participants did not complete a familiarization test before the testing phase as this would defeat the purpose of the statistical learning paradigm.

**Figure 2:**
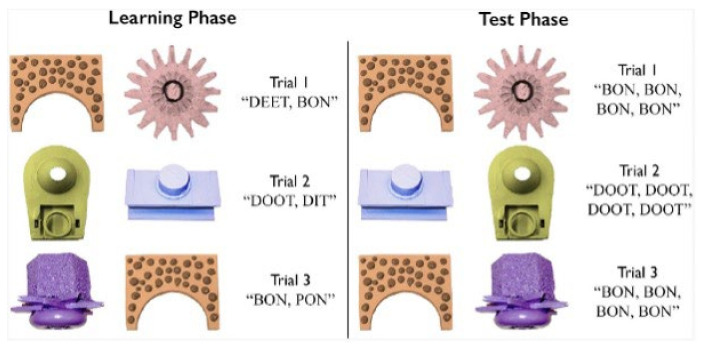
Example of a learning (**left**) and test (**right**) trial (figure from [27]).

Participants were tested directly after the learning phase and were told that they would view two images on the screen and would hear one word. They were instructed to press the left or right ALT key on the keyboard to indicate whether they thought the word corresponded to the left or right image, respectively. Trials in the testing phase used the same visual referent pairs as the learning phase, but the left and right designations of the images were randomized once (similar to [26]). In each trial participants heard four repetitions of the label corresponding to one image (the target word). The first token began 500 ms after presentation of the images, with 500 ms between tokens. Every word appeared as target word four or five times. As in the training phase, there were 36 trials in total with 24 nonMP trials, 6 cMP trials and 6 vMP trials. Trials were separated into three blocks of 12 trials, with block order counterbalanced between participants, and trial order within blocks randomized for each participant. Every trial lasted 6.5 s leading to a total test phase of approximately 4 min. An example of a learning and test trial is presented in Figure 2.

### 2.4. Statistical Analysis

The data were analyzed using the statistical program R [48] with the brms package (using Stan; [48,49,50]). We used a multilevel Bayesian regression model to analyze participants’ accuracy. We chose to use a Bayesian approach for the statistical analysis due to the advantage and flexibility of using probabilistic statistics with small sample sizes [51], as in the case of our L1 Mandarin L2 English group based in Sydney (N = 11). We have successfully used Bayesian modelling in previous studies where we provide further information and details of two other cases where probabilistic statistics are particularly useful [38,52,53,54].

For the multilevel Bayesian regression model reported below, we used dummy coding (the default in brms) for the factors of Language group and Pair type, with AusE and nonMP as reference levels. Approximate leave-one-out (LOO) cross-validation was used to find the best fitting models, which resulted in a model fitted with Language group and Pair type as fixed effects and Pair type and Trial number as random effects within-participants. We used weakly informative priors [55,56] with a Student-t’s distribution with 3 degrees of freedom, a mean of 0 and a standard deviation of 2.5. We used a Bernoulli distribution to model accuracy responses (which consist of either 0′s and 1′s).

After fitting the model, we proceeded with hypothesis testing. Based on the predictions from the L2LP model, we hypothesized that the L1 Mandarin L2 English groups would perform less accurately than the AusE group for vMPs and cMPs but not for nonMPs. If experience with living in a country were English is spoken and in particular the English variety of the stimuli influences performance, we also expected the L2 group based in Sydney to perform better than the group based in Shanghai. We quantified the evidence for the tested hypotheses by using evidence ratios (ER), which are used to assess the likelihood of the test hypothesis against its alternative. To test our hypotheses, we only consider ERs above 30 (or of 1/30 or beyond) which qualify as “very strong evidence” and ERs of 10–30 (or of 1/10–1/30) which qualify as “strong” evidence (see [57] as cited by [58]). For readers unfamiliar with Bayesian statistics, an ER of >19 is approximately equivalent to an alpha of 0.05 in frequentist null-hypothesis testing [59]. In addition to the ERs, we also report the hypotheses’ posterior probabilities (PP).

## 3. Results

### Accuracy

**Figure 3:**
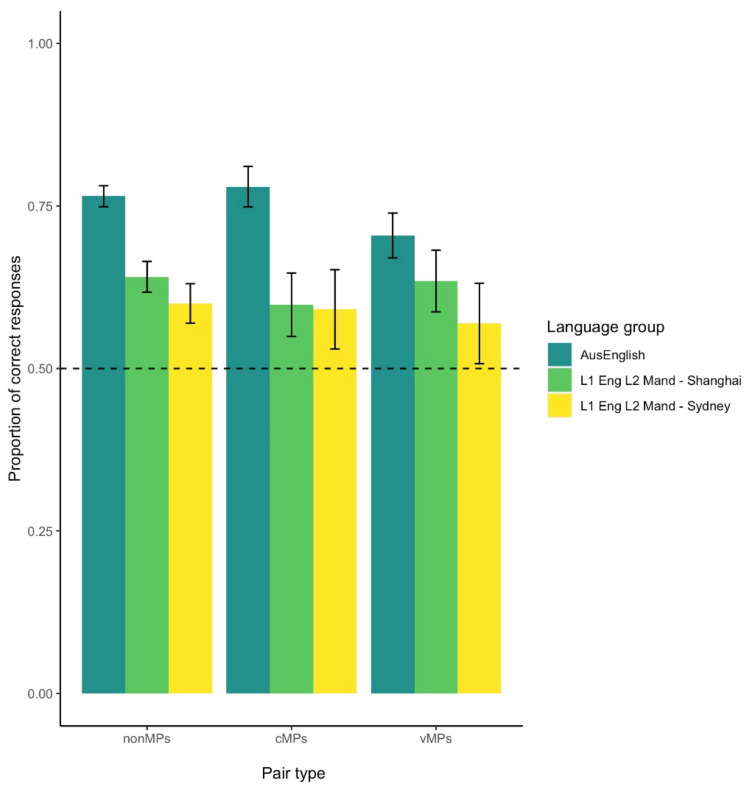
Mean accuracy in percentage per language group and pair type. Error bars indicate the standard error of the mean.

Figure 3 shows the mean accuracy responses per language group and pair type. We first analyzed participants’ accuracy (correct and incorrect responses) and tested whether participants were able to learn the word-object pairings for each pair type. Bayesian linear models on the Intercept, which are equivalent to frequentist one sample *t*-tests, revealed very strong evidence of above chance performance for all pair types in the AusE and in the L2 group from Shanghai, as indicated by posterior probabilities (PP) of > 0.98 and ERs of >3999. However, for the L2 group from Sydney we found very strong evidence of above chance performance only for the nonMPs (PP = > 0.999; ER = 3999), while evidence to support above chance performance was weaker for cMPs and vMPs (PPs = 0.92, 0.86; ERs = 11.82, 6.09, respectively). This is likely due to the higher response variability in this group (see Figure 3), which might be related to its smaller sample size (N = 11).

We then used a multilevel Bayesian regression model to estimate the interaction between Language group and Pair type on accuracy scores (see Table 1). We first tested whether there were differences in performance across the three pair types in each of the three language groups. For AusE, we found strong evidence that nonMPs and cMPs were more accurate than vMPs (PPs > 0.95; ERs of > 10), while no evidence for a difference between nonMPs and cMPs was found. These results replicate those reported in [26,27]) using frequentist statistics. Interestingly, no such difference between pair types was found for any of the L2 groups (PPs < 0.65 ERs < 10), suggesting their performance was similar across all three pair types.

The results in Table 1 indicate no performance differences between the two L2 groups (residing in Sydney or Shanghai), which was confirmed by further hypothesis testing where we found no evidence of a between group difference for nonMPs (PP = 0.40; ER = 0.68), cMPs (PP = 0.40; ER = 1.23) or vMPs (PP = 0.32; ER = 0.47). We thus combined the data from the two L2 groups into one L1 Mandarin L2 English group to test our hypothesis that the L1 Mandarin L2 English learners should have lower performance on vMPs than on cMPs or nonMPs because the vowels in the vMPs are not contrastive in their L1 Mandarin.

A second multilevel Bayesian regression model was run to estimate whether monolingual AusE learners (N = 26) indeed performed better than L1 Mandarin L2 English learners (N = 29). As shown in Table 2, we found very strong evidence (for nonMPs and cMPs) and strong evidence (for vMPs) that the AusE group had higher accuracy than the L2 group for all three pair types, which runs contrary to the hypotheses that these L2 learners will have lower performance for vMPs than the other two pair types and that they would only differ from monolingual English speakers in the vMP trials.

## 4. Discussion

This paper is the first to show that the mechanism of CSWL can be blocked by certain properties of the learner’s L1, which go beyond segmental differences between L1 and L2 vowels and consonants. Overall, participants learned the word-object pairings for all pair types. In line with AusE monolinguals tested in [27], AusE monolinguals here were best at identifying words in a nonMP or cMP context compared to a vMP context. However, contradicting our prediction, this pattern was not found for either L1 Mandarin L2 English group, who were less accurate than the AusE group for all three pair types. This suggests that the phoneme inventory differences between Mandarin and English do not explain their L2 word learning performance. Experience with AusE did not impact performance either, as both L2 groups performed similarly.

In contrast with [26,27], where simultaneous English-Mandarin bilinguals outperformed AusE monolinguals and L2 learners with diverse L1 backgrounds performed similarly to AuE monolinguals, here we found that L1 Mandarin L2 English had lower accuracy than AusE monolinguals. Prior research suggests that bilinguals have an advantage in pseudo word learning due to enhanced phonological memory [60,61,62,63] and executive functioning (e.g., [64]). Conversely, L2 learners have been found to have low sensitivity to L2 phonological contrasts that are absent in their L1 [65]. This may be explained by the idea that L2 learners perceive the sounds of a new language through their native phonological categories (e.g., [9,10,11,33,66], which can lead to L2 word recognition problems and L2 representations that continue to be L1-like [13,20,67,68,69,70,71]. Instead, simultaneous bilinguals are able to fully inhibit each of their languages selectively, while L2 learners, despite their proficiency, have trouble doing so. This has been shown many times in previous studies where language dominance yields to interference, especially for the L2 in sequential bilinguals [43]. For example, the pseudo words making up the vMPs of the present study included vowels that are not present in Mandarin, namely /ɪ/ and /ʊ/, as mentioned in the Introduction. As predicted by the L2LP model [11,13,14], such vowel contrasts are likely to be perceived as the closest acoustically related native vowel, leading to problems with the recognition of words containing those L2 contrasts, as has been shown for similar L2 word recognition cases (e.g., [13,20,21,22,23,69]).

However, absent or L1-like L2 representations in the L1 Mandarin L2 English group cannot explain the current results because they found all pseudo pair types equally difficult to recognize. Rather, we propose that their general word learning difficulty may have resulted from the specific stimuli presented to them. As mentioned in the Methods, participants heard pseudo words produced in infant-directed speech (IDS), which is a speech style often used by mothers and caregivers when speaking to babies and infants and contains more variable pitch relative to adult-directed speech (ADS) [41]. Although many studies have shown that IDS can be beneficial for word learning in infants [42,44] and adults [41,45,46,72], IDS might negatively impact word learning for listeners who have heighted attention to pitch variation, such as tonal language speakers for whom pitch variations signify different lexical items [73].

We propose that heightened discrimination of pitch variation may have resulted in L2LP’s Multiple Category Assimilation (MCA, L2LP; [74]), a scenario where an L2 category is acoustically similar to more than one L1 category, causing learners to perceive different tokens of a single L2 category as belonging to different categories in their L1 [17,22,66]. According to the L2LP proposal, this scenario results in listeners’ perception of contrasts that do not exist in the L2 [12] and is referred to as a SUBSET problem [11,74]. When L2 sounds are a subset of what the learner can actually hear, there is no overt information from the target L2 that would allow the learner to stop hearing the extra category or stop activating irrelevant or spurious lexical items [11,22,46,74] resulting in higher lexical competition and overall less efficient L2 lexicalization and recognition. It is likely that MCA plays a role in the overall lower performance of Mandarin speakers in this study, specifically due to the use of IDS for the stimuli tokens. The IDS-induced pitch variations may have resulted in L1 Mandarin L2 English learners’ perception of the two tokens of each word as two different words, challenging their ability to learn correct word-object pairs in the CSWL task. Importantly, the L2LP model is currently the only model of L2 perceptual and lexical developmental that can explain this type of L2 learning scenarios, starting from perceiving a single L2 category as more than one L1 category [75]. According to the L2LP model, this problem may not arise in simultaneous English-Mandarin bilinguals who may be able to de-activate their tonal language, succeeding at learning English words via CSWL and even surpassing monolingual English speakers, as reported in [26].

The L2LP explanation that the presence of additional pitch variation may be particularly problematic for speakers of a tonal language is further supported by findings that experience influences the perception of nonnative pitch variation. In many cases, tonal language experience is advantageous—for instance, Mandarin listeners outperform AusE listeners when learning a Thai tone distinction differing in pitch height contour [76,77]. In addition to tonal language learners, those who have experience with pitch via musical training have shown better tone discrimination [78], though not lexical tone learning [77]. However, in a recent study using the exact same CSWL paradigm and stimuli as in the present study [54] together with two standard music perception tests, we found that learners with high music perception abilities struggled most with IDS-produced words that had the highest pitch variability, i.e., vMPs. The tonal language speakers tested here struggled across all pair types, which may be due to them consistently using pitch information to discriminate between the exemplars of each word and across words for all pair types.

To confirm whether the additional pitch fluctuations induced by IDS indeed lead to a SUBSET problem and block CSWL in L1 Mandarin L2 English speakers, future research can use words produced in adult-directed-speech (ADS) with minimal pitch variation within and between exemplars of each word to examine whether word learning accuracy improves [54]. As vMPs naturally contain pitch variability, we expect the SUBSET problem to remain when tested with stimuli produced in ADS. If tonal language speakers indeed use suprasegmental information, such as pitch variations, when learning L2 words, their performance should thus improve more for nonMPs and cMPs than for vMPs when the variations in pitch are less prominent, as it is typical of ADS stimuli.

Lastly, we did not find a difference in experience with AusE, as both Mandarin groups performed similarly, providing no evidence that exposure to the L2 via an immersive environment mitigated L2 word learning difficulty. It could be that L2 exposure for the two L1 Mandarin groups is similar because while participants in Shanghai were not typically exposed to English in the community, both groups were students at English-speaking universities. Additionally, the two groups did not sufficiently differ in prior L2 exposure. However, a lack of group difference may also be due to a smaller sample size in the Sydney group, which resulted in higher variability in their results.

A limiting factor in this study is that by using self-reports as a measure of English proficiency, we may not be certain that participants have under- or overestimated their level of English. An English proficiency test administered alongside speech perception tasks may solve this issue. We also acknowledge that the missing details for each participants’ English proficiency is a limitation. However, with the available demographic data, we replicated previous results showing that individual background does not play a role in performance. In a future study, more detailed information should be collected to confirm that this variable indeed does not affect CSWL. It is also important to note that individual differences in cognitive abilities may influence word learning in such word learning paradigms and in previous cross-situational word learning studies. Results from our lab show that cognitive skills, such as visuospatial memory, inhibition, or flexibility were not significant predictors of cross-situational and incidental word learning in four-year-old children [79,80], but this may be different for adults.

## 5. Conclusions

To conclude, although L1 Mandarin L2 English learners were able to learn the pseudo English words in an ambiguous word learning scenario, their performance was overall lower than that of monolingual English speakers. Given that their performance was equally low for all pair types regardless of L1-L2 phonological relationships, an explanation solely based on the absence of L2 representations in this L2 learners cannot adequately account for the results. The more nuanced L2LP model’s explanation of a potential “subset problem,” in which these L2 leaners may have perceived different tokens of the same word as separate words because of their L1 tonal language background, seems to a more adequate and accurate account. However, further research is needed to confirm this proposal.

## Figures and Tables

**Table 1:** Hypothesis test results for the evidence ratios of the accuracy models’ interaction between pair type and language group.

Hypothesis	Mean	90% CI	ER	PP
*AusE*				
nonMP > cMP	0.10	[−0.24, 0.45]	2.17	0.68
nonMP > vMP	0.35	[0.01, 0.68]	22.05	0.96
cMP > vMP	0.45	[0.01, 0.89]	21.06	0.95
*L1 Mand L2 Eng–Shanghai*				
nonMP > cMP	0.21	[−0.19, 0.60]	4.32	0.81
nonMP > vMP	0.06	[−0.34, 0.45]	1.48	0.60
cMP > vMP	−0.15	[−0.66, 0.36]	0.45	0.31
*L1 Mand L2 Eng–Sydney*				
nonMP > cMP	0.05	[−0.45, 0.54]	1.30	0.57
nonMP > vMP	0.18	[−0.32, 0.67]	2.65	0.73
cMP > vMP	0.13	[−0.50, 0.77]	1.72	0.63

Note: Mean = mean of the effect’s posterior distribution. 90% CI = one-sided 90% credibility intervals. ER = evidence ratio = the odds that the effect is in the direction specified by the hypothesis. PP = the posterior probability of the tested hypothesis.

**Table 2:** Hypothesis test results for the evidence ratios of the second accuracy models’ interaction between pair type and language group.

Hypothesis AusEnglish > Mandarin	Mean	90% CI	ER	PP
nonMPs	0.78	[0.33, 1.22]	412.79	1.00
cMPs	1.03	[0.46, 1.60]	799.00	1.00
vMPs	0.51	[−0.03, 1.05]	15.20	0.94

Note: Mean = mean of the effect’s posterior distribution. 90% CI = one-sided 90% credibility intervals. ER = evidence ratio = the odds that the effect is in the direction specified by the hypothesis. PP = the posterior probability of the tested hypothesis.

## Data Availability

Anonymized data is available upon request to the first author.
